# A narrative review on diagnosis and treatment of ifosfamide-induced encephalopathy, the perspective of a EURACAN reference center for sarcomas

**DOI:** 10.3389/fphar.2025.1512966

**Published:** 2025-02-21

**Authors:** A. Torchia, S. Vari, C. E. Onesti, S. Ceddia, M. Maschio, G. Maggi, F. Riva, W. Faltyn, M. Russillo, V. Ferraresi

**Affiliations:** ^1^ Clinical and Molecular Medicine, Sapienza – Università di Roma, Rome, Italy; ^2^ Sarcomas and Rare Tumors Unit, IRCCS Regina Elena National Cancer Institute, Rome, Italy; ^3^ Center for Tumor-Related Epilepsy, UOSD Neuro-Oncology, IRCCS Regina Elena National Cancer Institute, Rome, Italy; ^4^ Psychology Unit, IRCCS Regina Elena National Cancer Institute, Rome, Italy; ^5^ Orthopaedic Oncology Unit, IRCCS Regina Elena National Cancer Institute, Rome, Italy

**Keywords:** ifosfamide, neurotoxicity, encephalopathy, sarcoma, treatment

## Abstract

Ifosfamide (IFO) is a nitrogen derivative used at different doses, alone or in combination, in the treatment of various types of solid and hematologic cancers. It is a pro-drug activated by cytochrome P450 enzymatic system into ifosforamide mustard, the alkylating component that carries out the cytotoxic effect of the IFO. The most common toxicities of IFO are gastrointestinal, cutaneous, hematological, urological, and neurological. The neurotoxicity may occur in up to 30% of patients and can manifest with a wide spectrum of clinical presentations (lethargy, somnolence, confusion, hallucinations, irritability, excitement, disorientation, weakness, seizures, movement disorders, coma) and a variety of EEG abnormalities, and is known as IFO-induced encephalopathy (IIE). There is no definitive explanation of the mechanism underlying this phenomenon, even though metabolism of IFO, which leads to the formation of neurotoxic components, is probably at the basis of neurotoxicity according to many hypotheses. Consequently, the different factors involved in IFO metabolism (i.e., genetic polymorphism of CYP2B6, GSTM1, GSTP1, and GSTT1; concomitant administration of drugs that affect the cytochrome P450 enzyme system; drug formulation) could be responsible of IIE. IIE is usually reversible by interrupting the IFO infusion and starting intravenous hydration but in some cases further interventions are needed. The most used pharmacological treatment is methylene blue, whose efficacy both as a curative and a prophylactic treatment has been the object of many studies, with mixed results. Other interventions that showed efficacy are thiamine (tested also as a prophylactic drug), dexmedetomidine, and hemodialysis. Other pharmaceuticals have been tested in a preclinical setting showing some activity: trifluoperazine, morin, caffeic acid phenethyl ester (CAPE), and alpha lipoic acid (ALA). The aim of this review is to gather the current knowledge about the mechanisms underlying the IIE and the current therapeutic approach and the future perspectives.

## 1 Introduction

Ifosfamide (IFO) is a chemotherapy medication used in the treatment of various types of cancer, including breast cancer, small cell lung cancer, testicular cancer, bladder cancer, non-small cell lung cancer, ovarian and cervical cancer, soft-tissue and bone sarcomas at different doses ranging from 50 mg/kg per day to 14 g/m^2^ per cycle. It is a pro-drug converted by cytochrome P450 (CYP) enzymatic system into its active metabolite, ifosforamide mustard, a cytotoxic alkylating agent. A side product of this reaction is acrolein, considered responsible for the hemorrhagic cystitis, a dose-limiting urotoxicity of IFO, prevented thanks to the antidote mesna (Kerbusch et al., 2001). The introduction of mesna allowed the safe administration of higher doses of IFO, e.g., in regimens used in the treatment of sarcomas (Gronchi et al., 2021; Strauss et al., 2021). The other most common adverse events (AEs) of IFO are nausea and vomiting, alopecia, blood cells count decrease, and central nervous system (CNS) toxicity in the form of a metabolic encephalopathy, known as IFO-induced encephalopathy (IIE) (Fan et al., 2015). The term encephalopathy is a generic definition indicating a disease in which the functioning of the brain is modified by some agent or condition. It comprises different conditions affecting the brain that, among the others, can be associated with chemotherapeutic agents. They can cause encephalopathy through different mechanisms including direct neurotoxicity, oxidative stress, blood-brain barrier disruption, and metabolic disorders (Barbosa-Azevedo et al., 2024). The first reports of neurological symptoms in patients treated with IFO date back to the 1970s and the 1980s, after the introduction of mesna (van Dyk et al., 1972; Cantwell and Harris, 1985; Meanwell CA. et al., 1986; Meanwell C. et al., 1986). IIE is among the most clinically relevant AEs of IFO, it can have various clinical presentations ranging from somnolence, mild mental confusion, or depressive periods to a state of hallucinations or coma. More specifically, IIE symptoms may include impaired consciousness, lethargy, somnolence, confusion, hallucinations, delusions, irritability, excitement, anxiety, disorientation, weakness, seizures, movement disorders, extrapyramidal symptoms, tremors, and coma (Curtin et al., 1991; Cerny and Küpfer, 1992; DiMaggio et al., 1994; Anderson and Tandon, 1991; Danesh et al., 1989), with a reported incidence of 10%–15% (Szabatura et al., 2015; Tajino et al., 2010). IIE is a clinical diagnosis with both early and late onset, usually within 48 h and up to 6 days from the start of the IFO infusion. There is no standard scale to define the severity of this kind of encephalopathy. Nevertheless, to date, it can be graded according to the National Cancer Institute Common terminology criteria for adverse events (NCI CTCAE) version 5, based on the severity of symptoms and their impact on activities of daily living (ADL). Furthermore, the Glasgow coma scale (GCS) is a simple and well-known tool that can be used to monitor the clinical development of patients over time (Reith et al., 2016). Symptoms of IIE are usually temporary. However, in some cases, IIE can be persistent and, rarely, fatal (Kerbusch et al., 2001; Yeager and Basnet, 2020; Watkin et al., 1989; Chain et al., 2022; Ataseven et al., 2021). In this narrative review we will discuss the etiopathogenesis of the IIE and its management in the clinical setting.

## 2 Etiopathogenesis

**FIGURE 1:**
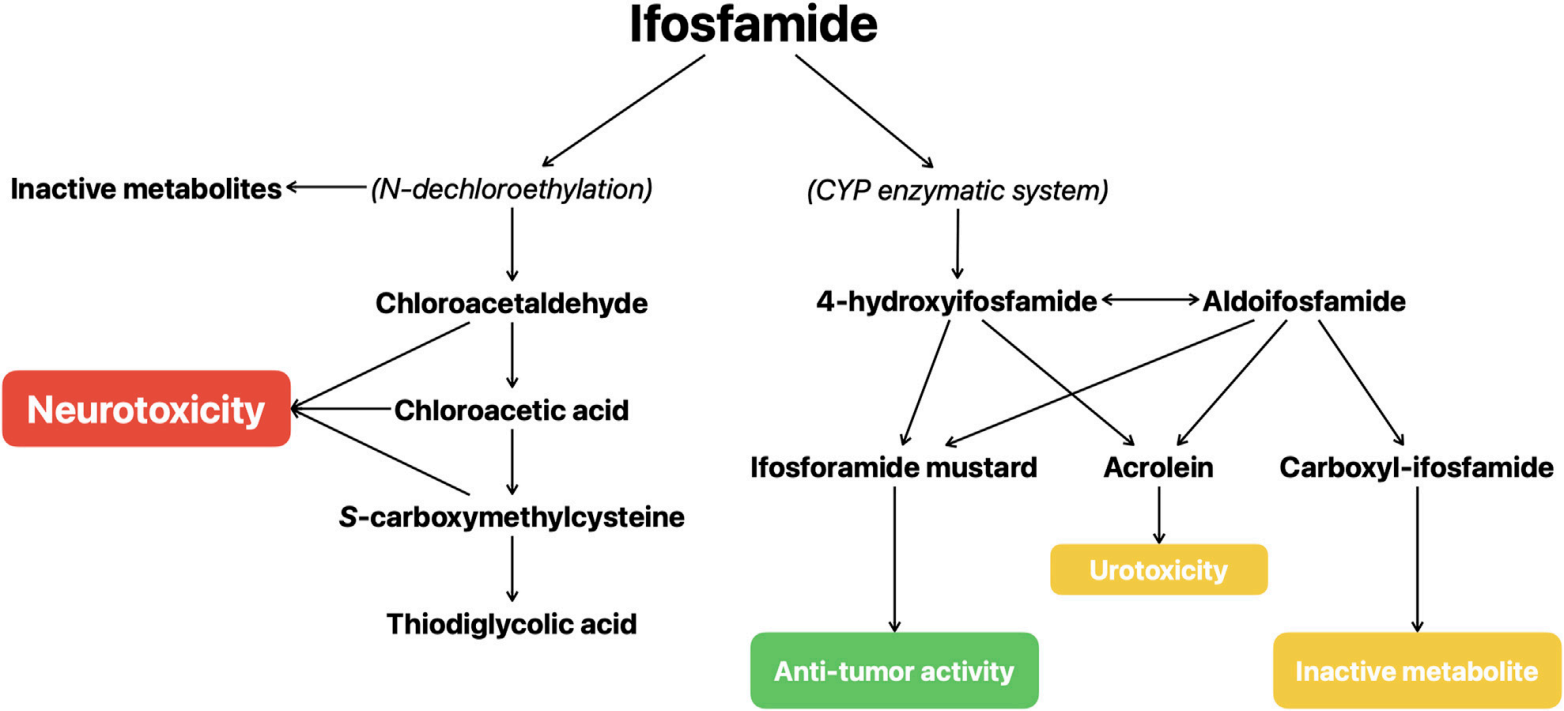
Schematic representation of the metabolism of ifosfamide.

The metabolism of the IFO seems to be central in the development of the IIE, as reported in a recent review by Idle and Beyoğlu (2023) focusing on the development and the metabolism of IFO. IFO is metabolized by the CYP enzymatic system, particularly by the CYP3A4 and CYP2B6, into the active metabolite (ifosforamide mustard) and various side products. IFO is converted to 4-hydroxyifosfamide (4-OH-IFO) from which ifosforamide mustard and acrolein are produced; 4-OH-IFO is in equilibrium with its tautomeric form, aldoifosfamide, that can be converted either into carboxyl-IFO (an inactive metabolite) and into acrolein and ifosforamide mustard. IFO can also undergo *N*-dechloroethylation reactions that lead to the formation of inactive metabolites and of chloroacetaldehyde (CAL), CAL could exert a neurotoxic effect through glutathione depletion, by influencing the mitochondrial terminal respiration chain, and through the formation of chloroacetic acid, a gluconeogenesis inhibitor, and *S*-carboxymethylcysteine, an agonist of alpha-amino-3-hydroxy-5-methyl-4-isoxazolepropionic acid (AMPA)/kainite receptors (Kerbusch et al., 2001; Storme et al., 2009; Huang et al., 2000; Lerch et al., 2006; Chatton et al., 2001; Yip et al., 2017) (Figure 1). Cyclophosphamide is closely related to IFO but is not associated with neurotoxicity and, unlike IFO, only a small proportion of it is metabolized through *N*-dechloroethylation, causing IFO to be associated to a greater increase of CAL concentrations (Li et al., 2010).

The administration of drugs metabolized by CYP3A4 and CYP2B6 may be associated with the IIE onset. Aprepitant and its pro-drug fosaprepitant, two selective antagonists of brain neurokinin 1 (NK1) with an antiemetic effect, are CYP3A4 moderate inhibitors with also a possible inductive effect (Sarcev et al., 2008; Shadle et al., 2004). Data from previous studies and case reports are mixed, showing a positive correlation between the concomitant administration of IFO and aprepitant (or fosaprepitant) and IIE in some studies, not confirmed in others (Séjourné et al., 2014; Durand et al., 2007; Shindorf et al., 2013; Patel et al., 2017; Jordan et al., 2011; Xiong et al., 2019). The interaction between these drugs has been the object of a systematic review from Vazirian et al. (2022), that included one randomized clinical trial (RCT) and eight retrospective cohort studies reporting a positive trend not reaching statistical significance between IIE and the concomitant use of IFO and aprepitant or fosaprepitant. However, the populations of the studies were highly heterogeneous with possible confounding factors and the association between the IIE and the administration of the said treatments were not always statistically evaluated. The concomitant administration of other drugs influencing the activity of CYP3A4 and CYP2B6, such as opioids, benzodiazepines, corticosteroids, and metoclopramide, was also investigated in a retrospective study by Szabatura et al. (2015). The study included 200 patients treated with IFO, of which 29 experienced IIE. The reported results show no effect of CYP3A4 inhibitors and substrates on IIE, and a statistically significant association between IIE and both opioids (odds ratio 2.81) and CYP2B6 inhibitors (odds ratio 5.17), despite a previous work suggested that the inhibition of the CYP2B6 pathway could be protective against IIE (Huang et al., 2000). Further data supporting the role of CYP2B6 derive from its genotyping performed by Duflot et al. on three pediatric patients experiencing IIE, reporting, in all 3 cases, the presence of loss-of-function variants (Duflot et al., 2018). An influence from variants of the genes of glutathione S-transferases (GST) was also hypothesized, albeit without a clear clinical significance (Zielińska et al., 2005).

Based on the neuropathological study of a patient who died due to IFO toxicity (including IIE) showing characteristics like those of Wernicke’s encephalopathy, Buesa et al. suggested that IFO and/or its metabolites could impair the function of thiamine and alter the cerebral glucose metabolism resulting in neuronal cell death (Buesa et al., 2003).

Other possible factors increasing the risk of IIE are impaired renal function, that could be caused by pelvic disease or previous administration of cisplatin, reducing the clearance of IFO and its metabolites; low albumin levels; hepatic disfunction and decreased bilirubin; acidosis; oral administration, shorter infusion time; central nervous system (CNS) metastases, previous CNS irradiation, pre-existent neurological disorders; electrolyte imbalance; both young and old age; female sex; obesity (Szabatura et al., 2015; Vazirian et al., 2022; Kettle et al., 2010; Lo et al., 2016; Howell et al., 2008). A recent review of the literature by Lee et al. (2020), summarizes the evidence on IIE risk factors, highlighting the insufficiency of data in this field, and the need for further research to establish the role of many suggested risk factors.

In a recent retrospective study on 172 sarcoma patients treated with IFO, Schmidt et al. showed a correlation between IIE and laboratory markers that can be associated with an inflammatory state, such as lower lymphocyte count, lower hemoglobin and calcium levels, elevated sodium, GGT and CRP levels, suggesting their potential utility in IIE prediction and diagnosis, being them routinely tested (Schmidt et al., 2022).

## 3 Neurological assessment

To date, there is no standardized approach for the assessment of IIE. Ideally, it should include both objective evidence of neurological deficits, and assessment of symptoms from a patient perspective, through neurological examination, neurophysiological parameters (EEG), patient-reported outcomes, and standardized evaluation scales. A pre-treatment neurological assessment could help to identify a pre-existing neurological dysfunction, that could increase the risk of neurotoxic adverse events from IFO. Likewise, an adequate assessment during the treatment is important to recognize the earliest signs of central nervous system toxicity, allowing a prompt intervention. Case reports describe EEG changes in patients with IIE (Müngen et al., 2022; Primavera et al., 2002; Feyissa and Tummala, 2014; Pavarana et al., 2005). The EEG alterations appeared during the acute phase of the encephalopathy, then gradually disappeared according to the clinical improvement of patients. A variety of abnormalities were recorded, comprising epileptiform discharges, background activity attenuation and slowing, and alterations consistent with non-convulsive status epilepticus (NCSE). Feyissa and Tummala (2014) evidenced that EEG could help identify patients with NCSE or those with epileptiform discharges who subsequently develop convulsive or non-convulsive seizures. In their study, the improvements in IIE symptoms after the interruption of the IFO infusion matched with the improved EEG changes upon repeated testing: resolution of electrographic seizures and epileptiform discharges and improvement in the background slowing. However, the results of a larger retrospective study by Gusdon et al. (2019) do not support this relation, instead suggesting that a marked background attenuation may be associated to poorer outcomes.

Conventional brain MRI could be useful to rule out other neurological conditions that may be responsible for the symptoms. However, there are no specific neuro-radiological findings associated with IIE. Literature data from other pathologies, especially hematological malignancies treated with chimeric antigen receptor (CAR) T cells, could be useful in the management of IIE. In a proof-of-concept study, Stoecklein et al. (2023) assessed the dysconnectivity index (DCI), based on functional MRI (fMRI) and resting state functional MRI (rsfMRI), in a small group of patients with lymphoma and melanoma during immune effector cell-associated neurotoxicity syndrome (ICANS) showing that higher DCI scores were associated with higher ICANS grades.

In the context of CAR T therapy, the American Society for Transplantation and Cellular Therapy (ASTCT) developed a new grading system for the immune effector cells-mediated central neurotoxicity, to address the lack of objectivity in the CTCAE reporting system and to stop relying on the evaluation of ADL, which can be difficult to assess in hospitalized patients. Their grading system uses the Immune Effector Cell-Associated Encephalopathy (ICE) score, derived from the CARTOX-10 (Neelapu et al., 2018), and a granular evaluation of key symptoms and signs such as depressed level of consciousness, seizures, motor disfunctions, and elevated intracranial pressure, aiming at objectively define the neurotoxicity (Lee et al., 2019).

## 4 Treatment

The management of IIE is essentially based on treatment discontinuation and hydration and there are no reference drugs with the specific indication. However, especially in case of severe toxicity, reversing agents are usually administered in clinical practice. The most used one is methylene blue (MB), administered intravenously at the dosage of 50 mg up to 6 times a day, whose effect is based on its activity as an electron acceptor, its ability to oxidate the excessive NADH formed through IFO metabolism and to inhibit the formation of CAA (Kerbusch et al., 2001; Patel, 2006; Küpfer et al., 1996). The rationale behind its efficacy in this setting was first showed by Küpfer et al. (1996) and then supported by case reports and reviews of the literature in the following years (Patel, 2006). Pelgrims et al. (2000) reported 12 patients with IIE, of whom eight were treated with MB infusion with full recovery after 24 h (4 patients), 48 and 72 h (2 patient, respectively); four patients did not receive MB and nevertheless recovered after 48 h. Turner et al. reported the cases of two patients treated with IFO and experiencing IIE, whose symptoms resolved after MB administration (Turner et al., 2003); Abahssain et al. (2021) reported four patients with IIE treated with MB, of which 3 showed a partial or total resolution of the neurological symptoms. They also conducted a review of the literature including 16 articles: 38 patients with IIE (65.5%) were treated with MB with a favorable response in 28 of them (75.6%). Despite its use in the clinical practice, there are no prospective randomized clinical trials evaluating its efficacy and safety in this setting and, whereas rare but potentially severe adverse reactions such as anaphylactic shock, Heinz body hemolytic anemia and serotonin syndrome have been reported, caution is needed (Vanhinsbergh et al., 2018; Sills and Zinkham, 1994; Snyder et al., 2017; Dewachter et al., 2005; Dewachter et al., 2011; Nubret et al., 2011). However, the use of MB in this setting can still be recommended based on the available data, considering the lack of established alternatives and the severity of the IIE.

Thiamine is another therapeutic option in the treatment of IIE, administered intravenously at the dosage of 100 mg every 4 h until symptoms resolution. The rationale for its use is based on the findings by Buesa et al. (2003) and on its favorable safety profile. Similarly to MB, data supporting the efficacy of thiamine in this setting comes from case reports. Buesa et al. (2003) reported the cases of 10 patients with IIE treated with thiamine with resolution of neurological symptoms such as low level of consciousness, confusion, hallucinations, anxiety, and asterixis. Hamadani and Awan (2006) reported three patients with IIE whose symptoms resolved after thiamine treatment within a mean time of 17 h. Ataseven et al. (2021) reported the case of a pediatric patient with IIE with severe clinical presentation treated with thiamine combined to MB whose neurological symptoms fully resolved. Müngen et al. (2022) reported a pediatric patient with severe symptoms from IIE successfully treated with thiamine to full recovery (Table 1).

**TABLE 1:** Reports on treatment of IIE with MB and thiamine.

	Author	Number of patients (pts)	Outcome	Notes
Methylene blue	Pelgrims et al.	12 pts with IIE 8 pts treated with MB	Received MB - 4 pts recovered within 24 h - 2 pts recovered within 48 h - 2 pts received within 72 h Did not receive MB - 4 pts recovered within 48 h	-
	Turner et al.	4 pts with IIE 2 pts treated with MB	Received MB: 2 pts recovered in 24 h Did not receive MB - 1 pt recovered within 24 h - 1 pt recovered (time unknown)	Symptoms included impaired consciousness, extrapyramidal symptoms, confusion, disorientation, nocturnal agitation, delusions, hallucinations, bizarre dreams, impaired sight Pts not treated with MB received it as prophylaxis before the subsequent cycle
	Abasshain et al.	4 pts with IIE treated with MB	3 pts recovered within 24 h; 1 pt died	Death due to malignancy progression
	Ataseven et al.	3 pts with IIE 1 pt treated with both MB and thiamine	Received MB and thiamine: 1 pt recovered in 2 weeks Did not receive MB and thiamine 2 pts recovered within 24 h	Pt treated with MB and thiamine was in a coma; patients not treated with MB and thiamine had seizures
Thiamine	Buesa et al.	10 pts with IIE treated with thiamine	10 pts recovered - 4 pts within 24 h - 4 pts within 48 h - 2 pts within 72 h	Symptoms included confusion, hallucinations, anxiety, asterixis, impaired consciousness
	Hamadani and Awan	3 pts with IIE treated with thiamine	2 pts recovered within 24 h, 1 pt within 36 h	Symptoms included confusion, disorientation, tremors, hallucinations, agitation, tremors; 1 pt was treated with MB before thiamine
	Ataseven et al.	3 pts with IIE 1 pt treated with both MB and thiamine	Received MB and thiamine: 1 pt recovered in 2 weeks Did not receive MB and thiamine: 2 pts recovered in 24 h	Pt treated with MB and thiamine was in a coma; patients not treated with MB and thiamine had seizures
	Müngen et al.	1 pt with IIE treated with thiamine	1 pt recovered within 24 h	Symptoms included impaired consciousness, agitation, disorientation, stupor

Blood purification therapy has been shown to decrease IFO concentrations both *in vitro* and clinical studies (Latcha et al., 2009; Sauer et al., 1990; Fiedler et al., 2001). Furthermore, it can decrease the concentration of potentially neurotoxic IFO metabolites such as CAA (Carlson et al., 1997). Based on these data and on the fact that impaired renal function is a risk factor for the development of IIE, dialysis has been successfully used to treat IIE, particularly in patients with severe clinical presentation, not responsive to MB and thiamine and with concomitant nephrotoxicity (Yeo and HaDuong, 2016; Nishimura et al., 2014; Cherry et al., 2013).

### 4.1 Prophylaxis

Prophylaxis should be considered in patients with an episode of IIE who continued the treatment with IFO. The most used agents are, similarly to the therapeutic setting, MB, and thiamine. The efficacy of MB was first reported by Küpfer et al. (1996), and it is commonly used in the clinical practice at the dosage of 50 mg up to every 6 h administered intravenously from the day before the start of IFO infusion. Thiamine is also administered in this setting, at the dosage of 100 mg every 6 h, alone or in combination with MB. However, there is limited evidence supporting their routine utilization, merely based on case reports and some retrospective studies (Buesa et al., 2003; Pelgrims et al., 2000; Turner et al., 2003; Hamadani and Awan, 2006; Kasper et al., 2004; Gharaibeh et al., 2019), contradicted by other retrospective studies that did not show any clinical benefit (Lentz et al., 2020; Richards et al., 2011).

Low albumin concentration is a potential risk factor for IIE. Albumin infusion as a preventive treatment has been investigated in a retrospective study with negative results (Kettle et al., 2010).

### 4.2 Preclinical data with other agents

Data from preclinical studies support the efficacy of other agents in counteracting the neurotoxic effect of IFO. Kiani et al. (2020) evaluated the use of trifluoperazine (TFP) in protecting isolated rat neurons against the damage of IFO. TFP is a typical antipsychotic drug that can also acts as an inhibitor of calmodulin, preserving the cell against the deleterious effects of calcium overload. TFP pretreatment in isolated rat neurons exposed to IFO reduced its cytotoxic effect. Çelik et al. (2020) evaluated the use of morin, a compound with anti-oxidant, anti-inflammatory, neuroprotective, anti-carcinogenic and antidiabetic properties, as a chemoprotective agent. The administration of morin in IFO-treated male rats was associated with enhanced antioxidant system, decreased cholinergic markers and inflammatory mediators, reduced mitochondria-dependent apoptosis, and other surrogates of neuronal damage. Ozturk et al. (2014) investigated the effect of alpha lipoic acid (ALA) against the IFO-induced neurotoxicity in rats, the rationale being the antioxidative properties of ALA. Their results showed that ALA has a protective effect against the IFO-induced neurotoxicity preserving the redox state of the cells and interfering with the apoptosis, induced by IFO. Ginis et al. achieved similar results investigating the effect of caffeic acid phenetyl estere (CAPA), a compound with antioxidative properties and able to interfere with apoptosis, in IFO-treated rats (Ginis et al., 2016).

## 5 Discussion

IFO neurotoxicity is a significant concern in the clinical management of patients undergoing chemotherapy with IFO. IIE can have various clinical presentations, ranging from transient somnolence to coma (Curtin et al., 1991; Cerny and Küpfer, 1992; DiMaggio et al., 1994; Anderson and Tandon, 1991; Danesh et al., 1989). Although its symptoms are usually mild and transient, IIE can have prolonged, severe, and sometimes fatal effects. The metabolism of IFO seems to be central in the etiopathogenesis of the neurotoxicity. Factors influencing its metabolism can however increase the risk of IIE, such as the concomitant administration of drugs influencing the activity of CYP3A4 and CYP2B6, impaired renal function, pelvic disease, previous administration of cisplatin, and low albumin levels (Vazirian et al., 2022; Duflot et al., 2018).

The management of IIE is based on treatment interruption and hydration and no drug has been proven to be effective. However, in the clinical practice, reversing agents as MB and thiamine are used in the more serious cases. Most of the data about their use in this setting comes from retrospective series and case reports and there are no prospective randomized clinical trials investigating their efficacy and safety. However, they still can be used to treat IIE based on the available data, considering the lack of alternatives and their favorable safety profiles. Both MB and thiamine are used in the clinical practice with a prophylactic intent, despite the low quality of the data about their efficacy, mostly derived from case reports (Buesa et al., 2003; Küpfer et al., 1996). Finally, both clinical and preclinical data support the use of hemodialysis in this setting, mostly in patients unresponsive to MB and thiamine treatment, consistently with the role of IFO metabolism and impaired renal function in the onset of IIE (Fiedler et al., 2001). Other agents, such as TFP, morin, ALA, and CAPA, have shown potential effectiveness against the neurotoxic effects of IFO in murine models. However, these compounds have only been studied in a preclinical setting and are still distant from a clinical application (Kiani et al., 2020; Çelik et al., 2020; Ozturk et al., 2014; Ginis et al., 2016).

Electroencephalography can be useful in the clinical management of patients with IIE. Different abnormalities were associated with the neurotoxic effect of IFO, usually following the clinical course of the patients. Moreover, there is data suggesting a correlation between the severity of IIE and specific EEG patterns, although not concordant. Thanks to its widespread availability and its non-invasiveness, EEG can be a useful tool in early detection of IIE, monitoring the clinical course of patients, and early identification of cases that can evolve to a serious presentation (Feyissa and Tummala, 2014; Gusdon et al., 2019).

There are unmet needs that should be addressed in the future. The routine use of MB and thiamine in clinical practice is supported by case reports and retrospective data, lacking prospective controlled studies. This issue should be addressed to get more certain data about their efficacy and their safety, also considering the possibility of rare but serious adverse events of these drugs, such as anaphylactic shock, Heinz body hemolytic anemia and serotonin syndrome. The diagnosis of IIE is essentially based upon clinical evaluation and can be challenging due to its heterogeneous and nonspecific presentations. In this context, objective and standardized methods should be developed specifically for IIE, including biomarkers, cognitive assessments, neuroimaging, and EEG. They would be fundamental for a more accurate and earlier diagnosis, and for a better management, helping identify those patients with a poorer outcome. In a recent retrospective study from Schmidt et al. (2022), a correlation between routinely tested inflammatory markers and IIE was shown, suggesting their potential clinical role as predictive factors of neurotoxicity.

Tools such as EEG and fMRI could be helpful to select those patients at a higher risk of developing the IIE, for an early diagnosis, critical for the prompt interruption of the IFO infusion, to monitor the clinical course, and to identify patients with a poorer prognosis (Feyissa and Tummala, 2014; Gusdon et al., 2019; Stoecklein et al., 2023). Due to the lack of objective diagnostic tools, the diagnosis of IIE is essentially clinical, therefore it is crucial to educate the patients and caregivers about the symptoms with which it can manifest. Together with the variability of clinical presentations, this can cause inconsistencies in the documentation of the IIE hampering both the treatment of patients in clinical practice and the comparison of different cases for research purposes. The adoption, or the adaptation, of objective scales and assessment tools, such as the grading system proposed by the ASTCT in the CAR T mediated neurotoxicity (Lee et al., 2019), can be useful for a more accurate and earlier diagnosis, and to better monitor the evolution of symptoms, allowing a more consistent treatment approach, and enabling a reliable evaluation of outcomes across different centers.

As a national and European reference center in EURACAN for the treatment of sarcomas, it is our intention to conduct a prospective study aimed at identifying possible predisposing factors for the development of IIE and establish recommendations on the prevention of neurotoxicity in at-risk patients. In our clinical practice, before administration of high-dose IFO, we currently perform a baseline neurological evaluation and an EEG to rule out predisposing neurological pathologies, to try to quantify the risk of neurotoxicity and provide for a more intensive symptom monitoring. We manage IIE cases mostly with MB, aside from interruption of the infusion and supportive hydration. If treatment resumption is possible, we administer prophylactic MB before the subsequent IFO infusions.

In summary, IIE remains a relevant clinical matter, with a potential impact on the clinical course of patients treated with IFO chemotherapy. Unmet needs remain, both in the diagnostic workup and the treatment, that should be addressed by further studies testing the efficacy and safety of drugs already commonly used in this setting, and the accuracy of diagnostic tools, providing higher-quality data supporting the daily clinical practice.
